# Coming to terms with a changing everyday life with dementia: What can we learn from people who are diagnosed while still working?

**DOI:** 10.1177/14713012251323939

**Published:** 2025-02-26

**Authors:** Louise Nygård, Ann-Charlotte Nedlund, Mervi Issakainen, Arlene Astell, Jenifer Boger, Anna Mäki-Petäjä-Leinonen, Ann-Louise Engvall, Birgit Heuchemer, Lena Rosenberg, Charlotta Ryd

**Affiliations:** Division of Occupational Therapy, NVS, 27106 Karolinska Institutet, Stockholm, Sweden; Department of Health, Medicine and Caring Sciences, Linköping University, Linköping, Sweden; Law School, Faculty of Social Sciences and Business Studies, 163043 University of Eastern Finland, Joensuu, Finland; University Health Network, Toronto, ON, Canada; Department of Psychology, University of Toronto, Toronto, ON, Canada; and Northumbria University, Newcastle upon Tyne, UK; Department of Systems Design Engineering, 8430 University of Waterloo, Waterloo, ON, Canada; Law School, Faculty of Social Sciences and Business Studies, 163043 University of Eastern Finland, Joensuu, Finland; Division of Occupational Therapy, NVS, 27106 Karolinska Institutet, Stockholm, Sweden; Division of Occupational Therapy, NVS, 27106 Karolinska Institutet, Stockholm, Sweden; Department of Rehabilitation, School of Health and Welfare, Jönköping University, Jönköping, Sweden; Division of Occupational Therapy, NVS, 27106 Karolinska Institutet, Stockholm, Sweden; 225341 Stockholm Gerontology Research Center, Stockholm, Sweden

**Keywords:** agency, citizenship, early onset, management, self-perception, subjective experiences

## Abstract

**Objective:**

The study’s aim was to better understand how persons, diagnosed with dementia while still working, strived to make sense of and come to terms with their changing everyday lives during the process of exiting work life.

**Methods:**

The study has an explorative, longitudinal design, following five persons who developed dementia while still working, with repeated, qualitative, in-depth interviews. Comparative analyses were combined with an interpretative approach, using the concepts doing, being, becoming and belonging.

**Results:**

Three overarching themes were created: i/Finding out an orientation to continued activity engagement, ii/ Relating to the diagnosis and available dementia specific activities, and iii/ Managing wellbeing and information related to health care. Findings illuminate how participants sought avenues for continued activity engagement in everyday life, based on their perceptions of what they were able to do, who they wanted to be and become, and where they felt they belonged.

**Conclusion:**

The participants’ agency came through strongly in their efforts to come to terms with changes in everyday life in their work and private lives, as well as with health care and dementia associations, underscoring that agency is vital and possible to support in persons with early-stage dementia.

## Introduction

Dementia has become an increasing concern in society, identified as one of the big challenges for health and wellbeing in the growing aging population worldwide (Cahill, 2020). Traditionally, dementia research has focused on the disabling consequences, taking ‘a deficit-focused bio-medical approach’ (O’Connor et al., 2022; Huizenga et al., 2022). However, a more nuanced image of dementia has started to emerge, where people living with dementia take a more active role in the research and are seen as citizens with their own abilities, interests, and rights rather than patients (Nedlund et al., 2019). This perspective is being strengthened by the increasing interest among researchers regarding how dementia might be experienced in very early stages, including people below the age of 65. In particular, the situation of people who develop dementia while still working – often called young-onset dementia – has received increased attention (Page et al., 2024; Smeets et al., 2024). Consequently, an expanded view is appearing in the literature, moving from a traditional care focus to a more multi-facetted view where people living with dementia might be co-workers, employees, or members of a community in a variety of capacities and roles. Yet, a shared, stereotypical view that dementia leads immediately to incapacity and loss of awareness (Low & Purwaningrum, 2020) seems to prevail. While early diagnosis is important for relevant actions, the devastating expectations from the diagnosis may also bring obstacles for persons with early onset dementia to make their voices heard and their agency recognized (Issakainen et al., 2023; Nygård et al., 2023); decisions may start being made based on stereotypes regarding dementia rather than on the individual and their specific context. This may be especially concerning for people who are still working, as the period around and after diagnosis often involves coming to terms with major changes in how everyday life is lived, with or without work (Page et al., 2024). This study set out to explore how persons who developed dementia while still engaged in paid work experienced their situation after onset and strived to come to terms with their changing everyday lives in the process of exiting vocational life.

### Literature review

The importance for people to engage in activities they find meaningful is well known, and the cognitively protective aspects of maintaining an active and engaged lifestyle for those with early-stage dementia have been put to the fore (Fallahpour et al., 2022). The medical recommendation following a dementia diagnosis has commonly been that the person should not work anymore, and the exit from work is often abrupt (Evans, 2019; McCulloch et al., 2016; Roach & Drummond, 2014; Williams et al., 2017). However, research suggests that leaving work is a lifechanging event, often experienced as a loss by persons with dementia, although for some it can come as a relief (Aspö et al., 2023; Ritchie et al., 2017; Roach & Drummond, 2014; Roach et al., 2016; Öhman et al., 2001). Such contradictory findings suggest the transition when exiting vocational life is a complex and multifaceted process that depends on the particulars of each individual person and their work context (Nygård et al., 2023). Earlier empirical studies pointed to the importance of having something meaningful to engage in after leaving employment due to dementia (Page et al., 2024). Yet, these persons are commonly left without support that could strengthen their individual resources and meet their needs for continued meaningful engagement in everyday life (Aspö et al., 2023; Issakainen et al., 2023).

So far, little is known about how people who develop dementia while still working manage to adjust to a changing everyday activity life, suggesting there is more to learn from the experiences of people with dementia who live through this process (Andrew et al., 2018). In a previous study, we focused on the vocational situation of five persons with dementia through explorations of the actions and decisions that were undertaken in their vocational lives, which included the views of their significant others (Nygård et al., 2023). These findings unpacked the influence of the common stereotypical understanding of dementia as immediately incapacitating the person, and strongly associated with old age, frailty and dependence: hence an alien in vocational life. The study exemplified how this view may trigger self-fulfilling expectations with decisive consequences for the persons. On the one hand, delay in diagnosis due to young age could lead to long processes of sick-leave and actions based upon false assumptions of ‘burn-out’. On the other, an early diagnosis generally led to leaving work, sometimes without considerations of the person’s capability or wish to continue working. A common theme was the importance of making sense of what is happening and having a say, i.e. acknowledging the person’s agency. But receiving a dementia diagnosis while still working does not only influence vocational life but brings about consequences to how life in general is lived, how days are occupied and changes managed. These topics have received little attention, and research grounded in individual cases has been called for (Andrew et al., 2018). In the present study, we continue the exploration of the unfolding experiences of the five persons with dementia, now widening the lens to embrace their everyday lives while transiting from work. Better insights into such transitions, including how they are experienced and managed, are needed to enable health care professionals to provide support with ‘planning for, initiating and maintaining post-retirement engagement in alternate or volunteer work, study, and new or existing hobbies.’ (Andrew et al., 2018, p. 14).

Moreover, according to Williams et al. (2017), a dementia diagnosis puts people at risk of being re-categorized by others to ‘someone who could no longer be thought of as a capable worker’ (p. 222). For some, this translates into perceptions of becoming unrecognizable; that is, by its influence on the person’s occupational capability, the dementia diagnosis can impact on how the person is perceived both by him/herself and others. This loss of occupational identity (Williams et al., 2017) can lead to loss of purpose, pride and self-worth, and a need to redefine self-perceptions of who one is. Such losses are not limited to work-life, but equally important in everyday life in the transition that usually follows a dementia diagnosis while relinquishing work roles (Andrew et al., 2018). In other words, a dementia diagnosis is likely to influence not only ‘what I can do’, but also perceptions of ‘who I am’, ‘who I will become’, and ‘where I belong’. The interacting dimensions of *doing*, *being*, *becoming* and *belonging*, originally introduced by Willcock (1999), have been found critical for understanding human experiences of engagement in activities and health in general, and hence also for the provision of appropriate support (Hitch et al., 2014a; 2014b). In short, engagement in everyday life activities ‘requires that we perform activities and occupations (*doing*) that meet the needs of both our selves (*being*) and others (*belonging*), that we can learn from and build upon through time (*becoming)*.’ (Hitch et al., 2014b, p. 12). Importantly, although all four dimensions simultaneous influence activity engagement, this influence is dynamic and in constant change, placing different dimensions in the foreground at different times in life, for example in the presence of a disabling diagnosis (Hitch et al., 2014b).

The relationship between peoples’ performance of activities – ‘doing’ - and sense of self – ‘being’ - has been extensively explored. Over time, the dimension of ‘becoming’ has received increasing attention, adding the person’s hopes, goals, and future aspirations, and more recently also ‘belonging’, i.e., the person’s sense of connectedness (Hitch et al., 2014a). We have chosen to use the phrasing *coming to terms with everyday life* to emphasise that the focus is on how these persons dealt with the activities and affairs of everyday life.

The specific aim of this study was to better understand how the persons, diagnosed with dementia while still working, strived to make sense of and come to terms with their changing everyday lives when dealing with the unfolding consequences brought about by the diagnosis in the process of exiting work life.

## Material and methods

### Design

This study has a longitudinal design, following five persons, who developed dementia while still working, with repeated qualitative interviews over time, taking an explorative stance (Merriam, 1998). With this approach, our intention was to stay as close as possible to the views of the persons living with dementia. In other words, the persons are seen as agents able to share their experiences and doing their best to deal with the evolving changes in everyday life.

### The Swedish context

In Sweden, memory investigations are initiated by general medical practitioners, but people below the age of 65 may be referred to a specialized memory clinic (Aspö et al., 2023). If the person is employed, the employer will be responsible for developing individually targeted actions to meet the employee’s needs after being diagnosed (Karlsson et al., 2014). However, this is up to each employer, and usually employees with early-stage dementia tend to experience a lack of understanding and support from employers, leading to abrupt exit from work without further actions in sight (Aspö et al., 2023).

### Sampling and recruitment

Sampling: Inclusion criteria (based on self-report) were as follows: i/ diagnosed with Mild Cognitive Impairment (MCI) or dementia or in the process of memory investigation with suspected dementia, ii/ working; employed or self-employed, or in paid work within the latest six months, iii/ sick-leave (full or part time) no more than six months, iv/ 50–75 years old, and v/ capable, willing and interested to take part in the longitudinal data gathering, including capable of giving consent. Ideally, we aimed for variation in the participants’ types of employment and work, length of sick-leave, education, age, and sex. However, because finding participants who met the second criteria turned out to be a challenge, we had to accept that variation in all criteria could not be fully achieved.

Recruitment: Participants were recruited through a variety of settings where we, based on our clinical experience, thought it would be possible to find persons that met our inclusion criteria. One participant was recruited through an out-patient unit for investigation of early cognitive impairment, two through a unit for investigation of driving capacity and two through a voluntary group for people with young onset dementia. Researchers presented the study at these facilities, and staff supported those persons with dementia who were potentially interested to establish contact with the researchers. Eventually, five participants, all of Swedish heritage, were recruited. We call the females Anna, Lisa and Maria, and the males Simon and Carl. Demographics and length of data collection for each participant are shown in Table 1. Each participant’s story is presented in-depth in Nygård et al. (2023).

**Table 1.** Demographics of participants and overview of data collection.

Given name and age	Living situation	Work % and employment or time since work cessation at inclusion	Diagnosis	Total number of interviews, period and length of data collection
Simon 55 years	Co-habiting	0% No employment One week	YOD* AD**	4 December 2018 – November 2019 (12 months)
Anna 60 years	Co-habiting	0% No employment Six months	YOD	4 February 2019 – February 2020 (13 months)
Lisa 53 years	Single	0% Permanent employment Six months	YOD AD	4 March 2019 – November 2019 (9 months)
Maria 58 years	Co-habiting	40% Permanent employment -	YOD	4 August 2019 – March 2021 (21 months)
Carl 71 years	Co-habiting	25%, Consultant -	AD	3 August 2019 – June 2020 (11 months)

YOD* Young onset dementia, AD** Alzheimer’s Disease.

Both verbal and written information was given to all participants before they gave consent to take part. Participants were repeatedly informed that they had the right to exit the study at any point without justifications We also repeatedly checked with each participant for their consent throughout the duration of the study. Approval was given by the regional ethical board (file number 2018/1313-31/5).

### Data gathering

All data were gathered through qualitative, in-depth interviews, framed through a general topic guide but also adapted to each participant’s unique situation (Brinkmann & Kvale, 2018). The situations that each person encountered were expected to be unique and continuously shifting. Hence, we aimed to follow each participant as long as some kind of transition - or reflections on transition - took place, expecting variation between participants. Consequently, what the challenges and changes were for each person and how these were dealt with would also be unique and dynamic (Merriam, 1998). As we followed each participant for around one year, they all faced changes in a variety of domains of everyday life, changes that they had to make sense of and deal with. All in all, Simon, Anna, Lisa and Maria participated in four interviews each, Carl three (in total 19 interviews, over 22 hours). Variations were due to each participant’s unique situation, and in part influenced by restrictions to meet during the Coronavirus Disease 2019 (COVID-19) pandemic. Eventually, data covered a mix of retrospective, present and prospective views: Maria and Carl still worked at inclusion (Carl worked also when data collection ended), Simon had stopped working only a week before the first interview, Anna and Lisa around six months before inclusion.

The interviews took place in the homes of participants in a relaxed atmosphere of conversation based on topics rather than standardized interview questions, starting off with the participants’ presentation of themselves in vocational as well as everyday life, what had happened related to their memory investigation or diagnostic procedures and how they experienced and made sense of this. In subsequent interviews, the interviewer followed each participant’s evolving experiences, with a particular interest in how their everyday lives developed in the presence of the diagnosis and its consequences, how they made sense of and dealt with these changes. The variation between participants and their evolving situation naturally influenced the development of later interviews. For example, reflecting in retrospect on the transition from working to sick-leave or retirement was relevant for some, while for others managing part time work as well as private life in the presence of a recent dementia diagnosis was in focus. The interviews strived to tune into the participants’ day-to-day adjustment and choices, as well as to their wishes, hopes and views of the future. Specific focus was also placed on participants’ self-initiated management approaches in different areas of daily life and how these were perceived (Nygård et al., 2022). All interviews were done one-by-one, and all were recorded and transcribed. The interviewers (co-authors and occupational therapists BH, A-LE and CR) have extensive clinical experience and interview training, but no prior relationship with participants. One researcher performed all interviews within each case and wrote memos after each interview session.

### Analyses

The analysis started during the data gathering process with continuous reflective meetings in the research team (Merriam, 1998). Through comparing data and our immediate understandings of potential meaning-making within and across participants, these discussions also influenced the subsequent interviews. When the data gathering process seemed to be nearly finished for all five, i.e., covering their recent and ongoing transition process, a focused comparative analysis started (Merriam, 1998). The first author thoroughly read all data, following the sequential order of the interviews with each participant. This reading included data-near coding of the transcribed interviews, guided by the aims of the study, i.e., an experience-near level (Borell et al., 2012). In this process, a particular interest was placed on each participant’s everyday life over the period around and after onset of dementia, and how they experienced, reflected on and acted in order to come to terms with their changing everyday lives. This comparative analysis resulted in three overarching themes: i/Finding out an orientation to continued activity engagement, ii/ Relating to the diagnosis and available dementia specific activities, and iii/ Managing wellbeing and information related to health care.

In parallel, we searched for a possible way to understand their experiences, sense-making and actions on a more synthesized and abstract level, inspired by hermeneutic principles, as applied through the Formal Data-Structure Analysis (FDA) (Borell et al., 2012; Gustavsson, 2000). This analytical step involved lifting individuals’ experiences to be embraced by a theoretical perspective. This abductive process means shifting between the experience-near perspective and a more experience-distance understanding (Borell et al., 2012). From this follows a back-and-forts process, moving between empirical data, emerging themes and possible ways of understanding on a more abstract level. To ensure rigor, this process included continuous discussions in the research team, reflecting on preunderstandings as well as on potentially contradicting data and competing ways to interpret data. The intention was to reach the most fruitful interpretation, i.e. it had to be grounded in most of the empirical data, and be the only one supported by most data, given the perspective taken (Gustavsson, 2000). As our focus was placed on the participants’ ways of coming to terms with the events and affairs of everyday life, we found the conceptual frame of *doing, being, becoming and belonging* (Willcock, 1999) to be enriching, adding a synthesized level of understanding their coming-to-terms-approaches.

## Findings

### Theme 1: Finding out an orientation to continued activity engagement

While the participants had similar views in terms of highly valuing keeping occupied, their take on coming to terms with and adjusting to the unexpected and unwanted turn imposed by the onset of dementia during their work-life showed more variation. Their ability to manage independently and to manage work also varied, and the next two sub-themes describe how they tried to discover their altering potential of doing and what this meant for their being, becoming, and belonging.

#### Finding out my abilities and adjusting to my altering potential to contribute

Finding out how they still were capable to contribute by doing seemed to be a key motivation for the participants’ efforts to maintain working as well as in finding other engagements. Maria, Anna**,** and Carl felt they did their utmost to maintain work, with adjustments in work tasks and work time. But continued work was also conditioned by or dependent on the quality the participants perceived their contributions held in relation to the requirements of the work tasks and other peoples’ judgement, exemplifying a key aspect of belonging. Carl, who still worked part time, said he was pleased to continue working for as long as his employer valued his contribution, which seemed to confirm to him that he belonged also in the employer’s eyes. Similarly, Anna told how she had trusted her supervisor at work to report at each weekly meeting how she had managed to perform. She explained: ‘…I had an agreement with them (the co-workers); you have to tell my boss if you think I don’t do well enough’.

It still caused Lisa great pain to talk about how worthless she had felt by ‘letting people down’ when she could not even manage to do the simplest, adapted work-tasks and hence ‘was let home’. This shows that she judged her work abilities and contributions to be inadequate, particularly as this, in turn, also influenced her co-workers’ accomplishments, suggesting that her established belonging in the work community was placed at risk. In contrast to Lisa, Simon explained that he had the opportunity to end his work life still being a successful person. After the diagnosis, he accepted an invitation to a new job, as he felt he still had more to give. When looking back, he felt this possibility had given him the chance to end work ‘with the flag up high’, he told. This seemed to support his continued sense of still being the same person, belonging in work life. Similarly, after eventually leaving work, Anna volunteered a few days a week in an organization as a resource person. To her, this duty was as serious as any job, and to her, it was utterly important to be able to contribute by ‘being useful’; it was central to her sense of belonging.

As exemplified above, the participants tried to find out how they could be useful and contribute by doing and thereby belong in a context, i.e., joining in tasks with others, suggesting the four dimensions are intrinsically interlinked. But while e.g. Anna’s, Carl’s and Simon’s cases exemplify how social contexts might support a person’s perception of being the kind of person one wishes to be and still belonging, Lisa’s case exemplifies the opposite; failures in doing at work also meant failing to meet one own’s standards of being, and no longer belonging.

#### Recognizing myself in the evolving situation

Over the course of the interviews, the participants reflected not only on their potential to contribute by doing at work and in other activities, implying belonging, but also on who they perceived themselves to be and potentially become, having to relate to changes following from dementia. While they perceived themselves as being – and wanting to remain - the same person, there were situations that challenged their usual notion of who they were. Consequently, maintaining the perception of ‘who I am’ required acknowledging and adjusting to the changes in doing, as doing also reflected ‘who I am’. This was often experienced as a challenge given the limitations that dementia imposed. For example, Lisa pondered on the challenge to learn to live with Alzheimer’s disease (AD), and thinking back to before the diagnosis, it seemed to be a different world to her. She wished to be ‘as usual’ but realised she was no longer able to be the person she used to be. ‘One has to learn again, new things, and that is difficult when one has Alzheimer’s and, in addition, has difficulty to renew oneself’, she explained. She struggled to accept and come to terms with new notions of who she had become, such as ‘being the one who needs support in everyday life instead of the one who takes care of everyone’, she told.

While Lisa’s reflections above exemplify her introspective view on who she had become, recognizing oneself was often reflected through the interaction with other people, and sometimes challenged by the shared, common-place image of dementia, i.e., who you might become once you have that diagnosis. For example, Simon said he still felt he was fundamentally the same person; he was himself, just with some memory issues and being somewhat slower. The only thing that had changed was that he no longer felt he was included as a full member of the family. ‘Somehow, I am not part of the family company to 100%, and no longer [hold] the sort of natural main role. Yes, it sounds weird when I put it into words, but… eeeh…. [I now have] more [of a] backseat role’, he said. That is, a subtle change in who he had become also impacted on his perception of belonging. His family now took precautions on his part: he reported that his wife had taken on a much stronger leadership role after his diagnosis, and the family did not always trust what he said and did. In other words: he had become a person that could not be trusted as before. This was nagging him a bit even if he understood the situation, he said.

All in all, it was obvious that their perception of ‘who I am’ (being) was closely related to contributing (doing) according to existing standards for a task or role at work or in family life. This was shown in reflections on how they viewed their own ‘being’ and ‘becoming’ as well as reflections on who they had become in the eyes of others. But doing also influenced belonging: to belong somewhere, the participants realized they also need to be able to contribute by doing and they tried to monitor changes in their ‘doing-ability’. Navigating this process, with a disabling disorder, could call for a new belonging while discovering changes in who they perceived themselves to be, or who others might perceive them to be; i.e. facing the risk of becoming an altered person beyond their recognition and choice. These intertwined aspects of doing, being, becoming and belonging are further elaborated in the next theme, focusing on the participants’ choices with respect to disclosure and available activities.

### Theme 2: Relating to the diagnosis and available dementia specific activities

The analysis revealed profound differences in the way the participants related to and acted upon the fact that they had dementia, and what the common image of dementia might mean to their perception of who they are, who they might become and where they could belong – in their own eyes as well as the eyes of others. As presented in two sub-themes below, the variety showed in their way of talking about their diagnosis in interviews as well as in their encounters with others, and in how they related to services and activities provided by authorities, health care and local voluntary associations.

#### Disclosing and talking about my dementia diagnosis

In our understanding, the participants shared a similar fear of becoming a ‘person with dementia’ according to the commonly held view, even though their approaches differed in terms of disclosure. At one end of a spectrum, Maria had chosen to keep her diagnosis a secret. At work, she let the others believe she suffered from so called ‘burnout syndrome’, and in her personal life, her diagnosis was disclosed only to a few close persons. This was very important to her; she was convinced that the label of dementia would make her become completely invisible in the eyes of others, lacking rights and protection, she said. No one will count on you anymore, she feared, i.e., she would no longer be regarded as able to join in and belong as a citizen as before the diagnosis. Maria explained that her standpoint had been further strengthened by prevalent scientific information about dementia: From lectures on its epidemiology and etiology, her impression was that people who get dementia are looked down upon as less educated, coming from poorer socio-economic contexts, having lived a poor life with unhealthy lifestyle habits, i.e., a population in which she did not want to belong. ‘If that is the picture that researchers are painting as typical of people with dementia, why would I tell anyone?’ she pondered.

At the other end of a spectrum, Carl felt that it was time to talk openly in a different way about what dementia might be. By explaining that one can live a decent life with the diagnosis, and showing it is possible to work, he rejected the common image of who you might become when you get dementia. ‘We have Alzheimer’s, and we can talk about it. We don’t look like monkeys, we are ordinary people,’ he said. He spoke openly about his diagnosis and noticed that some people did not really believe him as they could not see any signs of dementia in him, i.e., they confirmed that he still belonged to the population that he called ‘ordinary people’. By sketching an alternative image of dementia where he felt he could belong he wanted to influence the common view in society of who you become when you get dementia.

Exemplifying a position in between Maria and Carl, Simon also hesitated to disclose his diagnosis more openly than necessary, although he said that there was nothing risky or odd about it in everyday social life. Yet, Simon reported that neither he nor his wife felt they could tell authorities such as their bank about his health status when managing the financial consequences of exiting work. They feared that disclosure would influence how their interests would be taken into account as citizens, considering how society in general perceives a diagnosis of dementia and its impact on who you would become in their eyes.

#### Joining or rejecting organized activities for people with dementia

Being persons with dementia, the participants were invited to dementia-specific services, e.g., activities organized by local dementia associations, which suggested they would belong there. While such invitations in general were appreciated, they were received very differently. The participants’ responses varied accordingly, suggesting variations in their perceptions of where they might belong, given the diagnosis and its consequences. In fact, none of them recognized themselves in the common image of a person with dementia, but their approaches to come to terms with this alienation and the resulting issues of belonging differed, ranging from avoiding dementia specific contexts to using supportive activities for people with dementia as expected.

Both Maria and Simon avoided gatherings for people with dementia. Simon explained he felt these would make him feel he was sick. In contrast, he looked forward to the newly found contact with a neighbor family, who had shared the husband’s diagnosis in media. He hoped to gain more from sharing experiences with someone that still was relatively active and well, just as he was. In the last interview, he recounted that this had developed into ‘….a nice group of mates, without digging deep into our situations, you know. But sure, we also talk about, for example, how we tackle certain things, and that might concern very different situations’. Simon felt that such a social meeting with his neighbors offered him the opportunity to not change how he perceived himself. In contrast, he felt the association’s gathering defined the attendees by the diagnostic label being the only common theme.

In contrast to Maria and Simon, Lisa’s weekly activities were to a large part composed of group events at the dementia association. After some months, she announced that she had got day care at a specialized unit offering adapted activities; outings and lunches, gym exercise, and best of all in her view: weekly visits to a horse stable with the opportunity to actively engage in chores. In later interviews, she told she now felt at ease with no longer managing everything by herself. This acceptance had helped her to receive more help and support, and she no longer expressed any conflicts in perceptions of how these changes had influenced who she had become.

Moreover, alternative approaches appeared: When Carl was invited to the local dementia association’s meetings, he agreed to take the task on, he told, envisioning that he could contribute. But the meetings were horrifying in their sadness, he felt, with people crying and being very confused. He felt sorry for the others but did not identify with them, rather he identified with the group leaders. ‘I felt that someone has to ease the pressure here’, he said, and continued: ‘…so I became some kind of deputy leader and tried to get people to tell stories, and I initiated a variety of amusing topics to get them going’. Taking a leadership role seemed to confirm his perception of still being the same competent person as before, while also indicating that he did not belong in the group as a ’person with dementia’, but rather finding an alternative way of belonging. The following semester he felt the atmosphere had become a little brighter, but he chose to only take part when there were interesting lectures. His choices seemed to balance his perceptions of who he was and where he belonged. This exemplifies how persons with dementia might need to challenge the common view of who you become with dementia, and how this view is operationalized through the kind of engagement and support people with dementia are offered – in comparison to what they might feel is needed, as shown in the next theme.

### Theme 3: Managing wellbeing and information related to health care

The participants actively engaged in doing what they thought would best support their health and wellbeing, based upon ideas and information they had picked up from various sources. The diversity of efforts contributed to changes in how they spent their everyday lives. They also sought advice from health care, but received remarkable little response, suggesting a gap between their perception of still being agents able to act for their own best, and health care’s expectations of what people with dementia could do to maintain wellbeing, i.e., who a person would become with dementia.

#### Taking action and trusting it has an impact on my condition

According to the participants, it was important to take action in order to maintain best possible health and wellbeing. While they did this in different ways, responding to their unique conditions, their actions could be understood as reflecting their efforts to still be agents who take responsibility for their wellbeing by doing what they felt was best. For example, Carl repeatedly underscored how important it was to stay active, and he had adjusted his habits to include more healthy food, sleep, and exercise. While Anna had an exercise routine to walk at least 12 000 steps per day, preferably more, and enthusiastically used an app for cognitive training and games, Lisa stressed that her brain needed rest. Hence, Lisa was cautious to make sure that some days in her weekly routines were free. The calm life she experienced once getting used to not working also made her think about what made her happy now, she said. She strived for these moments, activities, and situations, recognizing that the person she had become experienced changed needs for wellbeing.

Maria told how she spent half of an average workday on taking care of herself, keeping updated on the disease, on hypotheses for causes and on treatment options. She also put great sums of money on alternative treatment substances, and she was very disappointed to find that health care neither asked persons with dementia about their diet and lifestyle habits, nor gave any advice on how to take care of your own health when living with dementia. Simon also pondered on how his own actions and attitudes might influence his situation. He was eager to do what he could to keep ‘well’ through sleep, exercise, and healthy diet, and remain being the same person for as long as possible, he said. Similar to Maria, he told he had not received any advice on how to best cope. ‘I guess they notice that I function and manage the situation, and then they see no reason to interrupt, it is just the medication then, how one deals with that’, he pondered.

All in all, by doing what they felt was best, the participants strived to influence their own wellbeing based on general advice, still being agents capable of taking action - but without support from health care to strengthen or guide their actions.

#### Feeling excluded from learning about my own condition

Another facet of the participants’ experiences related to their being, becoming, and belonging showed in their reflections on how health care and dementia associations treated their families differently from how they were treated as persons with dementia. The families had been invited to lectures and camps, to learn about dementia. Although education per se was valued, some participants reflected on how painful it was to be excluded as someone who is the object of education needs among family, the persons closest to you. This was most explicitly elaborated in Simon’s case: when discussing the altering roles in the family, he reflected on the strange situation when his wife and children went to the five education days about dementia, while he was not invited. He felt excluded and set aside, he said – there was nothing equivalent for him. When they returned home after each day of the course, he wanted to hear what had happened there, and asked: ‘What did you discuss about me today?’ Maria, who had persisted in asking for medical facts and advice, felt ignored as being difficult when the response from health care was ‘You ask so many questions!’ suggesting that she was not expected to ask for this information. Furthermore, Maria had also encountered the same attitude in the dementia association’s public information online. She told she had taken a fight with the association upon discovering that they address family first and foremost in their media. They had replied, she told, that she was not the intended audience, but assured they still think of her and all others with the diagnosis all the time.

These examples suggest that once diagnosed with dementia, participants were no longer expected to have and show agency by making efforts to influence their being or becoming. Instead, they felt they had trivialized to a target and object that their families needed education about.

## Discussion

In our findings, the themes illuminate commonalities as well as variations between participants’ approaches to come to terms with their changing everyday lives, while the multifaceted and generic nature of their sense making, i.e., the grounds for their approaches, and what these meant to them, became visible through the concepts of doing, being, becoming and belonging (Willcock, 1999). Findings revealed how the participants with dementia sought avenues for continued activity engagement in everyday life based on their perceptions of - and critical reflections on - what they were able to do or not, who they wanted to be and feared to become, and what these aspects meant to their sense of belonging. Thereby – and most importantly - the findings show the participants with dementia as active agents, that is; individuals who continuously seek to come to terms with changes and overcome problems they meet in any situation and context in everyday life, with and without work (Nygård et al., 2022). Further, while there could be variation in how participants strove to come to terms with their changing everyday lives, the framework of doing, being, becoming and belonging provides a way to understand individual variations as it may uncover the dynamic meaning-making behind their approaches in different realms of everyday life. This may help us to better understand their efforts beyond the traditional focus on capacity, and hence also how to support their efforts.

Among our participants, agency involved continuous reflections and efforts to find ones altering/new doing, being, becoming and belonging in everyday life and social relationships. Agency is not just an individualized trait but rather understood as interdependency (O’Connor et al., 2022). Interestingly, our findings exemplify how essential relational and interpersonal aspects might be for the person’s sense of self. This reveals how sensitive persons with dementia might be to the risk of becoming a person that they do not recognize, and the risk of not belonging – in their own eyes as well as in others. Other studies, for example Van Dijkhuizen et al. (2006), have argued that maintaining a sense of self in early-stage dementia depends on interactions with others. Our findings take this issue a step further by showing how participants did their best to maintain a sense of being the same person as before, through navigating contexts where they felt they still belonged given their altering potential to contribute, further exemplifying the presence of agency.

Moreover, the relational feature also stood out in our participants’ communication with health care. Even if they received advanced diagnostic investigations and pharmacological treatment, none of them received any formal support in managing the changes brought about by the diagnosis. This exemplifies that they perceived not being expected capability to influence their own state of wellbeing, that is; they had been deprived agency in the eyes of society. Yet, it has been emphasised that agency can be supported long into the progression of dementia (O’Connor et al., 2022), and our participants asked for such support. Recently, Aspö and colleagues (2023) described how persons diagnosed with young-onset dementia adopted strategies to remain in control, also including difficult decisions concerning disclosure as accessible information targeting them was missing. Earlier research has also shown that people living with dementia use a large variety of self-initiated management approaches to maintain a sense of control in everyday life (Nygård, 2004; Nygård et al., 2022). Hence, the findings of this study further underscore that it is vital to support the persons’ agency as shown by our participants’ efforts to do what they could to maintain wellbeing, and to find new activities and habits where they were able to do what was needed in order to belong.

Interestingly, with one exception, the participants often rejected activities offered by local dementia associations, as these seemed to fail to support their views of themselves (being): they did not feel they belonged there. Instead, they chose activities that allowed them to maintain a sense of being the same person, although mindful of changes in abilities, i.e., doing, and of potential consequences of such changes in fulfilling roles and tasks. Moreover, among some of the participants, only families received education about dementia while the person with dementia was excluded. They were not expected by health care or dementia agencies to try to influence their own wellbeing. This illustrates another feature of the dementia discourse; ‘stigma, discrimination and exclusion as a critical aspect of the dementia experience’ (O’Connor et al., 2022, p. 2342). Such a position taken by health care might further nourish family’s expectations of passivity on the part of the person with the diagnosis, causing frustration as exemplified in our participants. At the same time, research and media push older adults to take responsibility by living healthy lives with diets, exercise and cognitive stimulation to prevent disorders such as dementia (Fallahpour et al., 2022) – but once diagnosed, the expectations seem to be the opposite. If a person with dementia is excluded from information and feels being treated as an object that others need to manage, as exemplified among our participants, it is easy to understand hesitation to disclose the diagnosis. This could be a logical response to avoid further stigma, as Page et al. (2024) suggested, which might aggravate the person’s chances to make sense of and come to terms with what is happening.

Even though research increasingly has invited people living with dementia to share their views, the focus has often been on their perceptions of deficits and losses. However, if we view people living with dementia as agents with resources and a desire to influence their personal circumstances despite the diagnosis (Boyle, 2014), we can learn from them, and design support that is based upon their ways of management. To do that, the relational nature of the topic (O’Connor et al., 2022) must be acknowledged.

In our understanding, coming to terms with the evolving changes in everyday life in a variety of social practices and interactions in different contexts is a manifestation of applying one’s citizenship (Nedlund et al., 2019). This means that people’s everyday activities and affairs, however mundane and sometimes trivial they might seem, are of importance for their citizenship, thus linking their doings and perceptions of being, becoming and belonging (Willcock, 1999) to the wider lens of citizenship. All four dimensions are essential for practicing everyday citizenship (Nedlund et al., 2019). Our participants’ continuous reflections on the individual as well as the relational consequences of a dementia diagnosis (who I am, who I fear to become, and how that would influence my belonging) clearly show that we need to widen our perceptions when designing support for people with dementia, acknowledging the individual agency of the person with dementia in relational contexts; in relation to institutions, e.g. health care, as well as fellow citizens, e.g. co-workers. Not surprisingly, the relational feature of citizenship appeared as an overarching theme in O’Connor et al.’s review (2022), particularly emphasising citizenship as a social practice, and acknowledging the presence of active, everyday agency of people with dementia. Similar conclusions regarding the importance of reciprocal relationships, engagement in activities and belonging in community among people with early-stage dementia were drawn from a review by Huizenga et al. (2022). But O’Connor et al.’s review (2022) also underscores the importance of acknowledging issues related to identity and individual meaning-making among persons with dementia, suggesting the presence of a gap between the social and relational citizenship-lens and the individual self that needs to be bridged. We propose that the framework of doing, being, becoming and belonging can be a bridge that helps us better understand how to support coming-to-terms-approaches, i.e. agency, among persons with dementia. All in all, the citizenship lens acknowledges that there are lives to be lived also in the presence of a dementia diagnosis, which calls for better understanding of how *living well* with dementia might be facilitated, especially in the early stage of dementia (Huizenga et al., 2022).

### Methodological considerations

As always, generalisation of findings based on five persons’ experiences in a specific context requires great caution. To achieve depth, we used a longitudinal approach and added a theoretical and interpretative level of analysis. Ideally, our aim was to include participants who still were working and follow their transition while exiting work but finding persons in that situation turned out to be a challenge. In retrospect, the fact that some participants were looking back at, rather than living through, the immediate transition process of exiting work can be seen as a limitation, but also a possible strength because it might be difficult to reflect in the ongoing. The longitudinal approach opens up for understanding processes over time, but how do we know when it is time to end data gathering? Such decisions were taken based upon our understanding of data as well as pragmatical reasons, but it is indeed possible that continued data-gathering for longer time had added new insights.

## Conclusions

In conclusion, the study shows the participants as agents who strived to come to terms with their changing everyday lives caused by their dementia diagnosis by investigating their potential in doing, and what it brought about in terms of their being, becoming and belonging in everyday life; how they thought about and engaged in everyday citizenship. The participants asked for support that could strengthen their own actions towards wellbeing and strived to remain the persons they had been, persons who can influence their situation practicing both rights and obligations as citizens. In contrast, the attitudes and actions taken by the world around them – for example, in working life and services – seemed to expect them to be passive receivers, not asking questions or trying to influence their wellbeing. This seemed to apply particularly with respect to health care, further strengthened by the exclusion of the person with dementia from information and education directed to and given only to families. In other words, a clash seemed to appear between the participants’ images of, and actions taken to come to terms with, who they were, how they by doing could influence what became of them and where they belonged, and health care’s view: the latter seemed unprepared to meet their needs.

It is therefore critical that dementia associations as well as health care services elevate and position the voices of persons with dementia more powerfully, promoting their individualism, abilities, and autonomy, thereby acknowledging their rights as well as obligations while practicing everyday citizenship. Particularly in the early stages of dementia, we argue that such a point of departure would be especially decisive for provision of relevant support that could help persons to come to terms with the unfolding changes, in private everyday life as well as work. In short, society and support systems need to do a better job at enabling people with a dementia diagnosis to participate as persons with agency - as citizens - in the capacity that is right for them.
